# Acthaside: a new chromone derivative from *Acacia ataxacantha* and its biological activities

**DOI:** 10.1186/s12906-016-1489-y

**Published:** 2016-12-07

**Authors:** Abdou Madjid O. Amoussa, Mélanie Bourjot, Latifou Lagnika, Catherine Vonthron-Sénécheau, Ambaliou Sanni

**Affiliations:** 1 Unité de Biochimie et Biologie Moléculaire, Equipe de Biochimie et Substances Naturelles Bioactives, Faculté des Sciences et Techniques, Université d’Abomey-Calavi, 04 BP 0320, Cotonou, Benin; 2 Laboratoire d’Innovation Thérapeutique, Faculté de Pharmacie, UMR CNRS-Unistra 7200, 74 route du Rhin, CS 60024, 67401 Illkich, France

**Keywords:** *Acacia ataxacantha*, 7-hydroxy-2-methyl-6-[β-galactopyranosyl-propyl]-4H-Chromen-4-one (*acthaside*), Antimicrobial, Antioxidant

## Abstract

**Background:**

*Acacia ataxacantha* (Fabaceae), used in traditional medicine grows in the South-West of Bénin. Ethyl acetate extract of the barks of this species was previously reported to display various bioactivities, including antibacterial, antifungal and antioxidant activities. In the present study, we investigate the antimicrobial and antioxidant activities of compound isolated from ethyl acetate extract of *Acacia ataxacantha*.

**Methods:**

Purification, isolation and structural identification of isolated compound were done using various chromatographic and spectroscopic methods. Antimicrobial activity was investigated using a two-fold serial microdilution method. The inhibitory potency of isolated compound was evaluated by kinetic experiments. The antioxidant activity was also determined using 2, 2-diphenyl-1-picrylhydrazyl.

**Results:**

The isolated compound was identified as 7-hydroxy-2-methyl-6-[β-galactopyranosyl-propyl]-4H-chromen-4-one. As far as we know, this compound, named “*acthaside*”, reported for the first time, was active against all tested microorganisms with minimal inhibitory concentration ranging from 25 to 50 μg/ml. At 50 μl/ml, no growth was observed in almost all tested microbial after 24 h of exposure. The isolated compound had significant antioxidant activity with an IC_50_ value of 3.61 ± 0.12 μg/ml compared to quercetin (IC_50_ 1.04 ± 0.01 μg/ml).

**Conclusion:**

The present work demonstrates that the new chromen derivative isolated from *A. ataxacantha* may help treat bacterial and yeast infections. However, further studies are required to clarify the mechanism of action of this compound.

**Electronic supplementary material:**

The online version of this article (doi:10.1186/s12906-016-1489-y) contains supplementary material, which is available to authorized users.

## Background

Infectious diseases are the main cause of approximately one-half of all death in tropical countries. Some bacteria and fungi extremely pathogenic, are principal causes of these human infections. For several decades until now, the antibiotherapy has been one of the most important therapies used for fighting infectious diseases and has tremendously enhanced the health aspects of human life since its introduction. The discovery of antibiotics to combat these pathogens marked a revolution in the twentieth century [1]. However, the bacterial resistance is spreading throughout the world, inducing a steadily decreasing of relevant antibiotics [2]. The emergence of microbial resistance and the decrease in effectiveness of currently available antimicrobial agents requires the discovery for new antimicrobial substances with novel inhibitory mechanisms. Medicinal plants represent an alternative source for new antimicrobial agent discovery [3]. Medicinal plants have been used for centuries to treat infectious diseases and are considered as an obvious source of new antimicrobial compounds [4]. Numbers of bioactive metabolites have been isolated from these natural sources. According to the World Health Organization (WHO), medicinal plants could be the source of a variety of drugs. Antimicrobial properties of medicinal plant are being increasingly reported from different parts of the world [5]. The uneven availability, chemical diversity, structural complexity, lack of substantial toxic effects, and broad spectrum of antimicrobial activity of natural products, either as pure compounds or as standardized plant extracts, provide unlimited possibilities for new effective drugs and make them ideal candidates for new therapeutics [6, 7]. Members of the genus *Acacia*, which are widely distributed in tropical and subtropical regions, consist of about 1200 species [8], and are used medicinally for several purposes [9–12]. Previous phytochemical investigation on some species of the genus *Acacia* led to the isolation of flavonoids [13, 14], terpenoids [15] and polyphenols [16]. To the best of our knowledge, no phytochemical work has yet been done on *A. ataxacantha*. This species is widespread in much of sub-Saharan Africa. This species has been reported in Benin, Nigeria and Kenya for the treatment of tooth decay, dysentery, bronchitis, cough and joint pain [17–19]. Antimicrobial and antioxidant activities of the extracts of *A. ataxacantha* have also been reported previously [20, 21]. The lack of toxicity of the bark of this species was also reported [22]. These results suggest that this plant might contain bioactive compounds that act as antimicrobial and antioxidant agents. The present study were undertaken to evaluate antimicrobial and antioxidant properties of a new chromen derivative isolated from *A. ataxacantha*.

## Methods

### General experimental procedures

1D and 2D NMR spectra were recorded using Bruker 400 MHz spectrometer. The chemical shifts (δ) are reported in parts per million (*ppm*) relative to tetramethylsilane (TMS δ = 0). The coupling constants (*J*) are given in Hz. Deuterated methanol (CD3OD) was used as solvent in the NMR experiments. Mass spectral data ESI-MS, were recorded on Agilent technology instrument Accurate Mass Q-ToF spectrometer. The material used for chromatographic separation was Sephadex LH-20. Pre-coated silica gel 60 F_254_ TLC plates (Merck, Germany) were used for monitoring fractions and spots were detected with UV light (254 and 365 nm). Mueller Hinton (Agar and Broth) and Sabouraud Dextrose (Agar and Broth) were used for preparation of culture media for the antibacterial and antifungal activities respectively. Gentamicin, 2,2-diphenyl-1-picrylhydrazyl (DPPH) radical, and quercetin were purchased from Sigma-Aldrich Co., USA. All solvents used were of analytical grade.

### Collection and preparation of plant material

Fresh barks of *Acacia ataxacantha* were collected in September 2012 from Ouidah, Department of Atlantic, South of République du Bénin. Botanical identification was performed by a botanist from the Herbier National du Bénin at University of Abomey-Calavi and a specimen with voucher number AA 6509/HNB was deposited at the same Herbarium. The collected plant material was chopped into small pieces and air-dried in the laboratory (22 °C), under shade for two weeks. The dry plant material obtained was ground to a fine powder using an electric grinder (Excella mixer grinder).

### Preparation of plant extract

Dry powdered barks of *A. ataxacantha* (250 g) were successively extracted with hexanes (3 × 500 ml), dichloromethane (3 × 500 ml), ethyl acetate (3 × 500 ml) and methanol (3 × 500 ml) by maceration with continuous shaking (at 24 °C for 72 h) using an orbital shaker (Ika Ks 260 basic). The extracted matter from each solvent was filtered first using a cotton plug followed by Whatman No 1 filter paper. The resulting extracts were concentrated using rotary evaporator (BUCHI Rotavapor RII, Switzerland) under reduced pressure and extracts were stored in refrigerator at 4 °C.

### Isolation of bioactive compound

The best solvent system for column chromatography was selected for elution after carrying out the TLC of the ethyl acetate extract by the combinations of different solvents such as dichloromethane, ethyl acetate and methanol alternatively. Among all combination of solvents, dichloromethane: methanol combination showed the best resolution of the components of the extract on TLC plate.

The ethyl acetate extract (1 g) was dissolved in 1 ml of a mixture of CH_2_Cl_2_/CH_3_OH (50:50, v/v) and fractionated using Sephadex LH-20 column (diameter: 4 cm; Sephadex height: 25 cm). The elution solvent was the mixture CH_2_Cl_2_/CH_3_OH (50:50 and 20:80 v/v), to afford a total number of 31 fractions. A monitoring with TLC of these fractions yielded four main fractions (I-IV). Fraction III that consisted of major compounds in the extract was subjected to further purification. Purification of this fraction was done using preparative liquid chromatography (Gilson VP 250/21, Nucleodur 100-5 C_18_ec, Macherey-Nagel, UV detection 220 and 254 nm) by gradient elution (flow rate 20 ml/min) using water/acetonitrile (85:15 to 00:100 v/v) with 0.1% trifluoroacetic acid as mobile phase, to obtain compound 1 (9 mg).

### Structural elucidation of isolated compound

Structural determination of the isolated compound was carried out by spectrophotometric methods (1D and 2D NMR, mass and UV spectrometry). 1D and 2D NMR spectrum were recorded at room temperature with a Bruker NMR spectrometer 400 and 500 MHz in CD_3_OD. The 2D experiments (COSY, NOESY, HSQC, HMBC) were performed using standard Bruker programs.

### Acid hydrolysis of isolated compound

The isolated compound (1 mg) was dissolved in 6% HCl (1 ml) and heat at 80 °C for 2 h. The mixture was then extracted with CHCl_3_ (3 × 5 ml). The H_2_O phase was evaporated and dried to obtain the monosaccharide residue. This residue was identified as galactose by comparison with standards (galactose and glucose) on TLC in CHCl_3_/CH_3_OH/H_2_0 (8:5:1) [23].

### Microbial strains

The microorganisms used in this study included Gram-positives bacteria such as *Staphylococcus aureus* (ATCC 6538), *Staphylococcus epidermidis* (CIP8039), *Enterococcus faecalis* (ATCC 29212), *Staphylococcus aureus* Methicillin Resistant (SAMR) and Gram-negative *Pseudomonas aeruginosa* (CIP 82118). The microorganisms were obtained from Laboratoire de Biophotonique et Pharmacologie, University of Strasbourg, France. *Candida albicans* (CIP 4872) strain was obtained from national laboratory of drug control in Cotonou (Bénin). Bacterial cultures were maintained on Mueller-Hinton agar (MHA) and yeast cultures were maintained in Sabouraud Dextrose Agar (SDA) at 4 °C. Sub-culturing was done weekly. The bacteria were inoculated in Mueller-Hinton broth (MHB) for 18 h at 37 °C and yeast in Sabouraud Dextrose broth (SDB) for 48 h at 30 °C, prior to the test.

### Minimum inhibitory concentration

The two-fold serial microdilution method was used to determine the minimum inhibitory concentration (MIC) values of isolated compound against microorganisms [21]. The stock solution (1 mg/ml) was prepared by solubilizing 1 mg of isolated compound in 50 μl of dimethyl sulfoxide (DMSO 2.5%) followed by 950 μl of MHB. Briefly, 100 μl of isolated compound (100 μg/ml), gentamicin and fluconazole (50 μg/ml) were two-fold serially diluted with Mueller-Hinton broth for antibacterial assay and Sabouraud broth for yeast assay in 96-well microplates to make eight concentrations of isolated compound (100-0.78 μg/ml) and control (50-0.39 μg/ml). 100 μl of freshly culture of bacteria (10^6^ CFU/ml) and yeast (2 × 10^5^ CFU/ml) was added to each well. DMSO (2.5%) was used as negative control while gentamicin and fluconazole were used as positive controls. The microplates were covered and incubated at 37 °C. After 18 h of incubation, 40 μl of 0.2 mg/ml solution of p-iodonitrotetrazolium (INT) which is an indicator solution for determination of bacterial growth were added to each well and microplates were further incubated at 37 °C. The minimal inhibitory concentration was determined 30 min after addition of INT.

### Minimum bactericidal and fungicidal concentration

The minimum bactericidal concentration (MBC) and minimum fungicidal concentration (MFC) of isolated compound was determined according to the method of Escalona-Arranz et al. [24]. To determine the MBC and MFC, aliquots of 20 μl from all dilutions not showing any growth of bacteria and yeast were inoculated on sterile MHA plates (for bacteria) and SDA (for yeast) by spreading using swab sticks. Inoculated plates were incubated at 37 °C for 24 h for all bacteria while those inoculated with fungi were incubated at 30 °C for 48 h. After incubation, the concentration at which there is no visible growth of the organisms on the agar plates was recorded as the minimal bactericidal concentration (MBC) for bacteria and minimal fungicidal concentration (MFC) for yeast. The experiment was carried out in triplicate.

### Determination of MIC index

The MIC index (MBC/MIC) was calculated for isolated compounds and positive controls to determine whether a compound had bactericidal/fungicidal (MBC/MIC ≤4) or bacteriostatic/fungistatic (4 < MBC/MIC <32) effect on the growth of bacteria or fungi [25].

### Time-kill kinetic index

The time-kill kinetic index of isolated compound was determined as described by Miyasaki et al. [26] with slight modifications. The objective of this test is to know the duration of bactericidal or fungicidal effect of isolated molecule. Briefly, bacterial and yeast overnight cultures were diluted to the 10^6^ CFU/mL with MH and SD broth respectively. Equal volume of each diluted inoculum and tested compound were mixed at their respective predetermined MBC and MFC values and incubated with shaking at respective temperature of 37 °C for bacteria and 30 °C for yeast. At different time intervals viz. 0, 1, 4, 8, 12, … 36 h, 0.1 mL of the mixed suspension was spread on suitable agar petri dishes in triplicate and incubated for 36 h at suitable temperature. After 18 h incubation, viable colonies were enumerated. The results were recorded in terms of log_10_ CFU and plotted vs. time for each microbial tested.

### In vitro antioxidant activity

The antioxidant activity of isolated compound on the stable radical 2,2-diphenyl-1-picrylhydrazyl (DPPH) was performed using the method developed by Danielle and Lall, [27] with slight modifications. In this assay, 96 wells plates were used. For this assay, the stock solution was prepared by disolving 2 mg of isolated compound in 1 ml of methanol HPLC-grade to obtained 100 μg/ml. Quercetin was also prepared at the same concentration as the standard reference. Briefly, 200 μl of stock solution and quercetin was added separately to the wells in the top row. This was followed by the addition of 100 μl of methanol to the remaining wells. From the wells of the first row of the plate, the two-fold serially dilutions were performed to obtain a concentration ranges from 1.56 to 100 μg/ml. Finaly, 200 μl of methanolic solution of 2,2-diphenyl-1-picrylhydrazyl (2%) were introduced in each well of a microplate. The plates were allowed to develop in the dark room for 30 min before the measurement of the absorbance at 517 nm using a Microplate Reader (Rayto-6500). The capability of the compound and the reference standard to scavenging the free radical was determined following formula bellow:

$$ \mathrm{Inhibition}\ \mathrm{percentage}\ \left(\mathrm{I}\%\right) = \left[\left({\mathrm{A}}_{\mathrm{Blank}}\hbox{--}\ {\mathrm{A}}_{\mathrm{sample}}\right)\ /\ {\mathrm{A}}_{\mathrm{Blank}}\right]\ \mathrm{x}\ 100, $$

Where, A_Blank_ is the absorbance of the control reaction (containing all reagents except the test sample) and A_sample_ is the absorbance of sample (isolated compound or quercetin).

The concentration of samples reducing 50% of free radical DPPH (IC_50_) was determined using the regression line representing the inhibition percentage of DPPH versus the sample concentration. The assay was replicated three times and results are expressed as mean ± standard deviation.

### Statistical analysis

The results were expressed as means of triplicate determination ± standard deviation (SD). The graphical was performed using the Graph Pad Prism 6.1 software (Microsoft, USA).

## Results and discussion

In our previous work, the ethyl acetate extract of bark of *Acacia ataxacantha* was found to have significant activity against *S. aureus* (ATCC 6538), *S. epidermidis* (CIP 8039), *E. faecalis* (ATCC 29212), *S. aureus* Methicillin Resistant (SARM) and *P. aeruginosa* (CIP 82118). Ethyl acetate extract was active against all bacteria with the minimum inhibitory concentrations values of 325 μg/ml against *S. aureus* and 625 μg/ml against all other tested bacteria [20]. The phytochemical analysis of this active extract led to the isolation of a chromen derivative. The structural identification of isolated compound was elucidated using a combination of spectroscopic methods. Biological activity of this compound was also evaluated.

### Structural elucidation of isolated compound

**Fig. 1:**
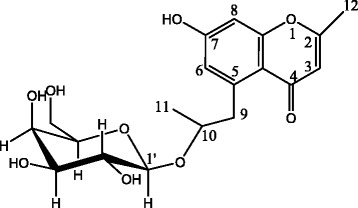
^1^H-^1^H COSY correlations of isolated compound

**Fig. 2:**
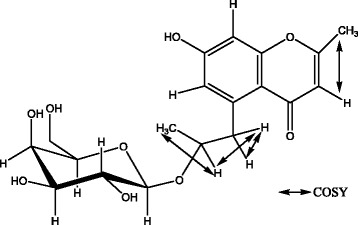
HMBC correlation of isolated compound

**Fig. 3:**
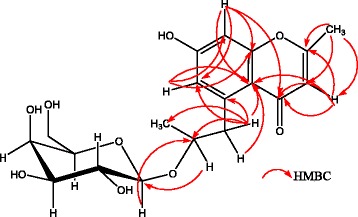
Structure of 7-hydroxy-2-methyl-6-[-galactopyranosyl-propyl]-4H-Chromen-4-one (*acthaside*), a new chromen derivative from the bark of *Acacia ataxacantha*

The compound was isolated as white amorphous powder which showed a violet-blue fluorescence in UV-365 nm with KOH reagent on thin layer chromatogram. The Mass spectra measured with LC-ESI-MS positive mode showed a molecular ion at m/z 396.14203 [M + H]^+^ (calcd for C_19_H_24_O_9_, 396.14243). Its also showed UV (CH_3_OH) λmax 225, 245 and 288 nm. The ^1^H and ^13^C NMR spectra (Table 1) of isolated compound included resonances corresponding to aromatic and glycosidic protons and carbons (Table 1). The ^1^H NMR spectrum of the compound in CD_3_OD showed two methyl at δ_H_ 2.25 (3H, d, *J* = 0.7 Hz) and 1.09 (3H, dt, *J* = 6.2, 3.6 Hz), two oxymethylene protons at δ_H_ 2.97 (1H, dd, *J* = 12.1, 7.4Hz) and δ_H_ 3.68 (1H, dd, *J* = 12.1, 5.3Hz) and one anomeric proton at δ_H_ 4.45 (1H, d, *J* = 7.8 Hz) correlated in the HSQC spectrum with one anomeric carbon at δ_C_ 103.0 in the ^13^C NMR spectrum. The ^13^C NMR spectrum of isolated compound showed 19 carbon signals, of which six were assigned to the sugar moieties and the remaining 13 to the aglycone. The COSY and ^1^H spectra of isolated compound also showed two aromatic protons at δ_H_ 6.65 and 6.62 (each 1H, d, *J* = 2.4 Hz) assignable to H-6 and H-8 respectively (Fig. 1). The HSQC spectrum allowed to correlate these protons to carbons signal at δ_C_ 119.8 (C-6) and δ_C_ 102.6 (C-8). Complete assignment of the remaining resonances of the aglycone in the ^13^C NMR spectrum was achieved by analysis of the HSQC and HMBC data, which confirmed the presence of 7-hydroxy-2-methyl-6-propyl-4H-Chromen-4-one. Furthermore, one anomeric proton resonance corresponding to O-linked sugar was displayed in the ^1^H NMR spectrum as one doublet at *δ*_H_ 4.45 (*J* = 7.8 Hz) which correlate in HSQC to an anomeric carbon at *δ*_C-1_ 103.0. Acid hydrolysis of isolated compound led to identified galactose, by HPLC comparison with a standard. The NOESY spectrum showed NOE correlations between H-1′ and H-10, H2-9. These results led to identify a β-D-galactopyranose as described previously [28, 29]. A correlation between H-1′-gal and *δ*_C_ 76.9 in the HMBC spectrum of isolated compound specifies C-10 of the aglycone as the site of O-glycosylation (Fig. 2). Thus, the structure of isolated compound was elucidated as 7-hydroxy-2-methyl-6-[β-galactopyranosyl-propyl]-4H-Chromen-4-one (Fig. 3). As far as we know, this compound is a chromen derivative which has not been reported previously (Additional file 1).

**Table 1:** NMR Spectroscopic data for *acthaside*

Position	Isolated compound (*acthaside*)	
	δ_H_ (J in Hz)	δ_C,_ type
1	**-**	**-**
2	-	166.7, C
3	5.94, (d, 0.7)	111.6, CH
4	-	181.7, C
4a	-	115.3, C
5	-	144.0, C
6	6.65 (d, 2.4)	119.8, CH
7	-	161.8, C
8	6.62 (d, 2.4)	102.6, CH
8a	-	163.1, C
9	2.97 (dd, 12.1, 7.4) 3.68 (dd, 12.1, 5.3)	43.3, CH_2_
10	4.06 (m)	76.9, CH
11	1.09 (d, 6.2)	21.5, CH_3_
12	2.25 (d, 0.7)	19.9, CH_3_
1′	4.45 (d, 8.0)	103.0, CH
2′	3.07 (dd, 8.7, 8.0)	75.5, CH
3′	3.27 (m)	78.3, CH
4′	3.20 (m)	71.9, CH
5′	3.21 (m)	77.9, CH
6′	3.58 (m) 3.75 (dd, 11.8, 1.6)	62.9, CH_2_

### Antimicrobial activity of isolated compound

The isolated compound was evaluated in vitro for its antimicrobial activity and the results are presented in Table 2. Compound was tested for antimicrobial activity by determining MIC, MBC and MFC against four Gram-positive bacteria: *Staphylococcus aureus* ATCC 6538, *Staphylococcus epidermidis* CIP 8039, *Enterococcus faecalis* ATCC 29212, *Staphylococcus aureus* Methicillin Resistant, one Gram-negative bacteria: *Pseudomonas aeruginosa* CIP 82118 and a fungi: *Candida albicans* CIP 4872. The tested compound exhibited various degrees of antimicrobial effect against the tested microorganisms. Many reports consider the antimicrobial activities to be significant if the MIC is ≤ 10 μg/ml, moderate if 10 < MIC ≤ 100 μg/ml and low if MIC > 100 μg/ml [30, 31]. The tested compound had moderate antibacterial activity against five test bacteria with MIC and MBC ranging from 25 to 50 μg/ml. The isolated compound had also moderate antifungal activity against *C. albicans with* MIC and MFC values of 25 and 50 μg/ml. *Staphylococcus epidermidis* and *C. albicans* were the most sensitive with the MIC value of 25 μg/ml. The mechanism of antibiosis of the isolated compound was calculated using MIC index to elucidate whether the observed antibacterial effects were bactericidal or bacteriostatic [25]. The isolated compound showed the same values of MIC and MBC against *S. aureus*, *MRSA*, *S. epidermidis*, *E. faecalis* and *P. aeruginosa*. The same values obtained for MIC and MBC indicate the bactericidal nature of isolated compound against these bacteria. The isolated compound also showed a fungicidal effect against *Candida albicans* (MIC = MFC). For decades ago, the search for antimicrobials was oriented towards the discovery of natural compounds that can inhibit Gram-positive and gram-negative, which are most often responsible for infectious diseases. Gram-positive bacteria cause a broad spectrum of disease in immunocompetent and immunocompromised hosts. Despite increasing knowledge about resistance transmission patterns and new antibiotics, these organisms continue to cause significant morbidity and mortality, especially in the health care setting [32]. The Gram-negative cell wall (made up of lipopolysaccharide) is complex and multilayered structure, which makes access to membrane more restricted and barrier to many environmental substances including synthetic and natural antibiotics [33]. The results of this study indicate that the *acthaside*, isolated from *A. ataxacantha,* could be an agent able to cross this tough barrier. Our results have also shown that the tested compound was active against *C. albicans*, suggesting its possible use in the treatment of fungal infections.

**Table 2:** Minimum inhibitory concentration (MIC) and Minimum bactericidal and fungicidal concentrations (MBC, MFC) of *acthaside* (μg/ml) from *A. ataxacantha* against microorganisms

	Minimum inhibitory concentrations (μg/ml)					
Microorganisms^a^	Gram (+) bacteria				Gram (-) bacteria	Yeast
	*S.a*	*S.a.m.r*	*S. ep*	*E.f*	*P.a*	*C. a*
Isolated compound^b^	50	50	25	50	50	25
Gentamicin	0.39	0.39	0.78	0.39	0.78	Nt
Fluconazole	Nt	Nt	Nt	Nt	Nt	0.78
Minimum bactericidal and fungicidal (μg/ml)						
Isolated compound^b^	50	50	25	50	50	25
Gentamicin	0.78	1.56	1.56	0.78	1.56	Nt
Fluconazole	Nt	Nt	Nt	Nt	Nt	1.56
MIC index						
Isolated compound	1	1	1	1	1	1
Gentamicin	2	4	2	2	2	Nt
Fluconazole	Nt	Nt	Nt	Nt	Nt	2

^a^ *S. a : Staphylococcus aureus; S.a.m.r : Staphylococcus aureus* methicillin resitant*; S.ep : Staphylococcus epidermidis*; *E. f: Enterococcus faecalis; P. a: Pseudomonas aeruginosa; C. a: Candida albicans. Nt* not tested

^b^isolated compound: 7-hydroxy-2-methyl-6-[β-galactopyranosyl-propyl]-4H-Chromen-4-one (*acthaside*)

### Time-kill kinetic study

**Fig. 4:**
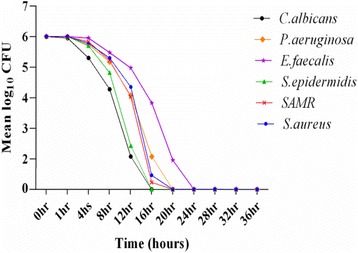
Time-Kill kinetic analysis of *acthaside* against tested baterial and yeast

Previous researchers have used MICs and MBC as prediction tools for antimicrobial action of plant extracts or pure compounds, thus limiting the use of such data since it does not consider time-related antimicrobial effects, such as killing rate [34]. The kill kinetics provides more accurate description of antimicrobial agents than does the MIC [35]. In this study, we explored the time-kill activity of *acthaside* isolated from the barks of *A. ataxacantha* against *S. aureus*, *SAMR, S. epidermidis*, *E. faecalis*, *P. aeruginosa* and *C. albicans.* The rate of microbial killing after exposure to the MBC or MFC of isolated compound was summarized in Fig. 4. The time required to achieve a reduction of 3log10 CFU is an acceptable index of bactericidal or fungicidal activity of analyzing time-kill [36]. The results showed that no tested organisms showed significant bactericidal or fungicidal activity in the first hour. However, 3log_10_ reduction in viability of all tested microorganism was observed after 16 h of exposure. More interesting, the 3log_10_ CFU of *C. albicans* and *S. epidermidis* was reduced to zero after 16 h exposure. The 3log_10_ CFU of all others tested bacterial was reduced to zero after 20 h of exposure except *E. faecalis* (24 h). According to some authors, an antimicrobial agent is a “substance that kills or inhibits the development and the multiplication of microorganisms, such as bacteria, fungi, protozoa or viruses” [37, 38]. Among numerous pure compounds having this feature, *acthaside* could be considered. This antimicrobial power of *acthaside*, could be due to the presence of benzopyran ring (chromen) in its structure. Chromens represent a class of secondary metabolites that have generated great attention because of their interesting biological and pharmacological properties [39]. In additional, the new chromen derivative isolated from *A. ataxacantha* shows broad spectrum of antimicrobial activity against Gram-positive and Gram-negative bacteria and yeast (*C.albicans*). These results corroborate with the work of Charles et al. [40] which showed that uvafzelin (a chromen derivative), isolated from the stems of *Uvaria ufielii* has presented a broad spectrum of antimicrobial activity against Gram-positive and acid-fast bacteria. A key feature is that the lipophilic nature of the chromen derivatives helps to cross the cell membrane easily [41].

### Antioxidant activity

**Fig. 5:**
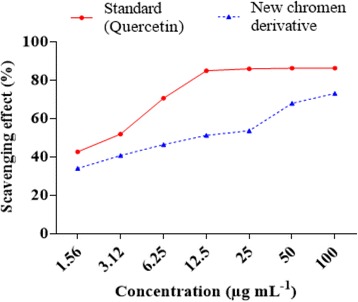
Scavenging effect (%) of 7-hydroxy-2-methyl-6-[β-galactopyranosyl-propyl]-4H- Chromen-4-one and Quercetin (standard) on DPPH radical

The hydrogen-donating activity measured using DPPH radicals as hydrogen acceptor, showed dose-dependent activity. The results were compared to quercetin (Fig. 5). From 1.56 to 12.5 μg/ml the scavenging activity of isolated compound varied from 34.03 to 51.32% compared to the control (42 .71 to 84.93%). From 25 to 100 μg/ml, the percentage of inhibition of DPPH radical varied from 53.67 to 73.12% for isolated compound and from 85.94 to 86.28% for the control (quercetin). The concentration of antioxidant required for 50% scavenging (IC_50_) of DPPH radicals was also determined. The IC_50_ value, is a parameter widely used to measure antioxidant activity. The new chromene derivative isolated from *A. ataxacantha* had interesting antioxidant activity with an IC_50_ of 3.61 ± 0.12 μg/ml compared to quercetin (IC_50_ 1.04 ± 0.01 μg/ml). The antioxidant activity of this compound could be assigned to its phenolic nature. The presence of electron-donating substituents such as -OH, on the chromen core could also be responsible of intersting radical scavenging activity. Thus, this compound would reduce free radicals and then prevent cell damage. The antioxidant power of this new compound would justify its antimicrobial broad-spectrum activity against Gram-positive and Gram-negative bacteria and against yeast (*C. albicans*). It has been demonstrated, an increase in the antimicrobial activity of pure compounds when they are combined with antioxidants [42]. Therefore, we consider that if a compound has antimicrobial and antioxidant properties, this dual effect could enhance its antimicrobial activity.

## Conclusion

The present research work through a systematic chemical and biological investigation has determined and identified for the first time the compound 7-hydroxy-2-methyl-6-[β-galacto-propyl]-4H-chromen-4-one (*acthaside*) as a new antimicrobial substance and antioxidant, isolated from *A. ataxacantha*. This study demonstrated that the isolated compound present a broad spectrum antimicrobial activities against Gram-positive and Gram-negative bacteria and also against *C. albicans* (yeast). The isolated compound has also showed a promising antioxidant activity when compared with standard drugs. The results obtained in the present study showed the interest of the ethnopharmacological and chemotaxonomic approaches in the search for active substances against the microbial infections. The active tested compound may, either to be a model for the synthesis of more active and less toxic analogues or to be used in association with antimicrobial commercial drug, as they may reverse the resistance of microbial to antimicrobial drugs. However whether the new compound isolated should act as an effective therapeutic agent, the study of its mechanism of action would be necessary before application.

## Additional file

Additional file 1: NMR and MS spectrum of the new chromone derivative (Acthaside). (PDF 439 kb)

## Abbreviations

1D: Mono dimensional
2D: Bidimensional
ATCC: American type culture collection
CFU: Colony-forming unit
CH_3_OH: Methanol
CIP: Collection Institute Pasteur
COSY: Correlation spectroscopy
DCM: Dichloromethane
DMSO: Dimethyl sulfoxide
DPPH: 2,2-diphenyl-1-picrylhydrazyl
EtoAc: Ethyl acetate
HMBC: Heteronuclear multiple bond correlation
HPLC: High performance liquid chromatography
HSQC: Heteronuclear single quantum coherence spectroscopy
INT: p-Iodonitrotetrazolium
MBC: Minimum bactericidal concentration
MFC: Minimum fungicidal concentration
MHA: Mueller-Hinton agar
MIC: Minimum inhibitory concentration
NMR: Nuclear magnetic resonance
NOESY: Nuclear overhauser effect spectroscopy
SDA: Sabouraud dextrose agar
TFA: Trifluoroacetic acid
TLC: Thin layer chromatography
