# Supplementary material for: Acthaside: a new chromone derivative from Acacia ataxacantha and its biological activities
Source: BMC Complement Altern Med. 2016 Dec 7;16:506. doi: 10.1186/s12906-016-1489-y (PMC5142280; doi:10.1186/s12906-016-1489-y)

NMR and MS spectrum of the isolated compound (*Acthaside*)

^{1}H NMR

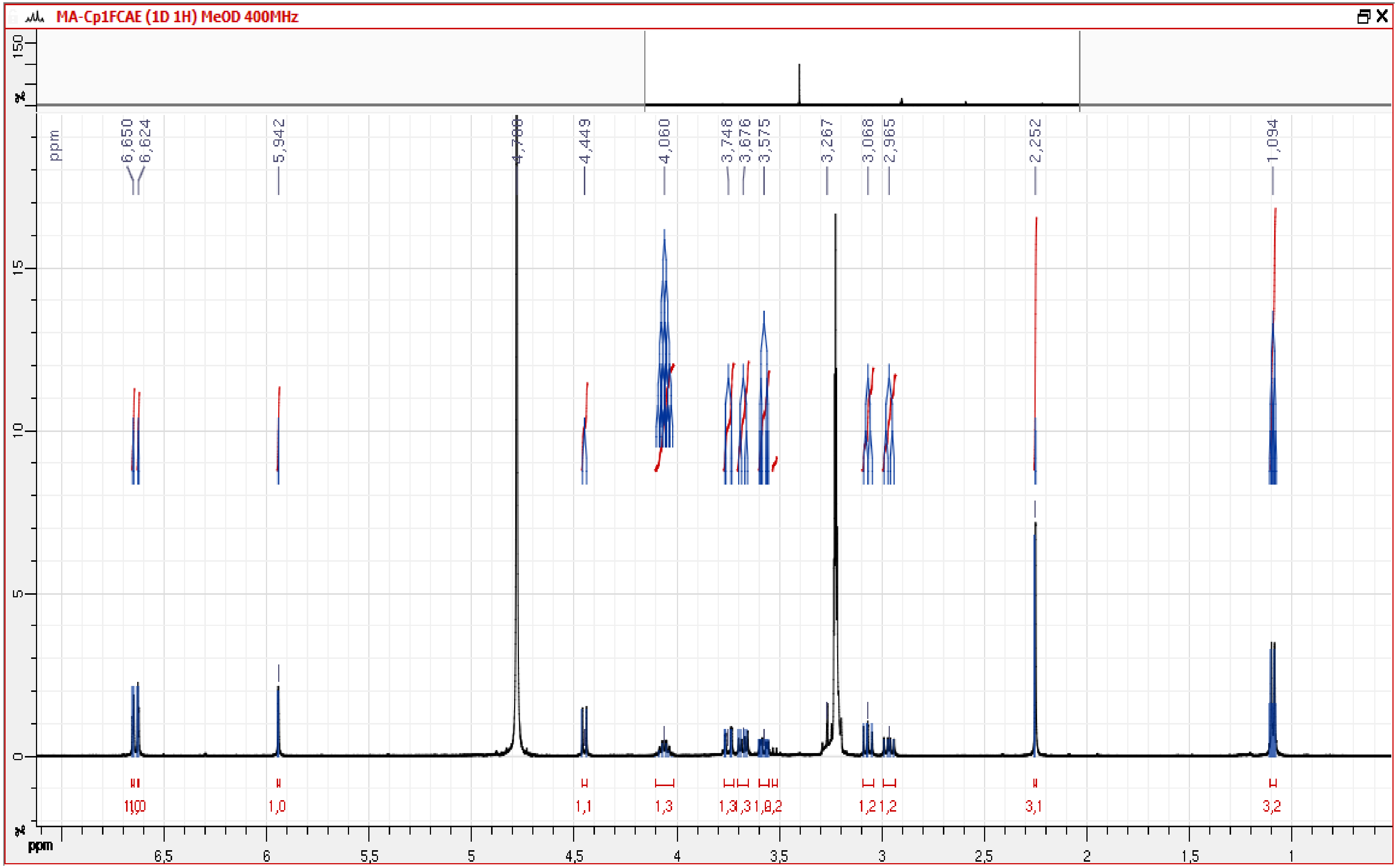

^{13}C NMR

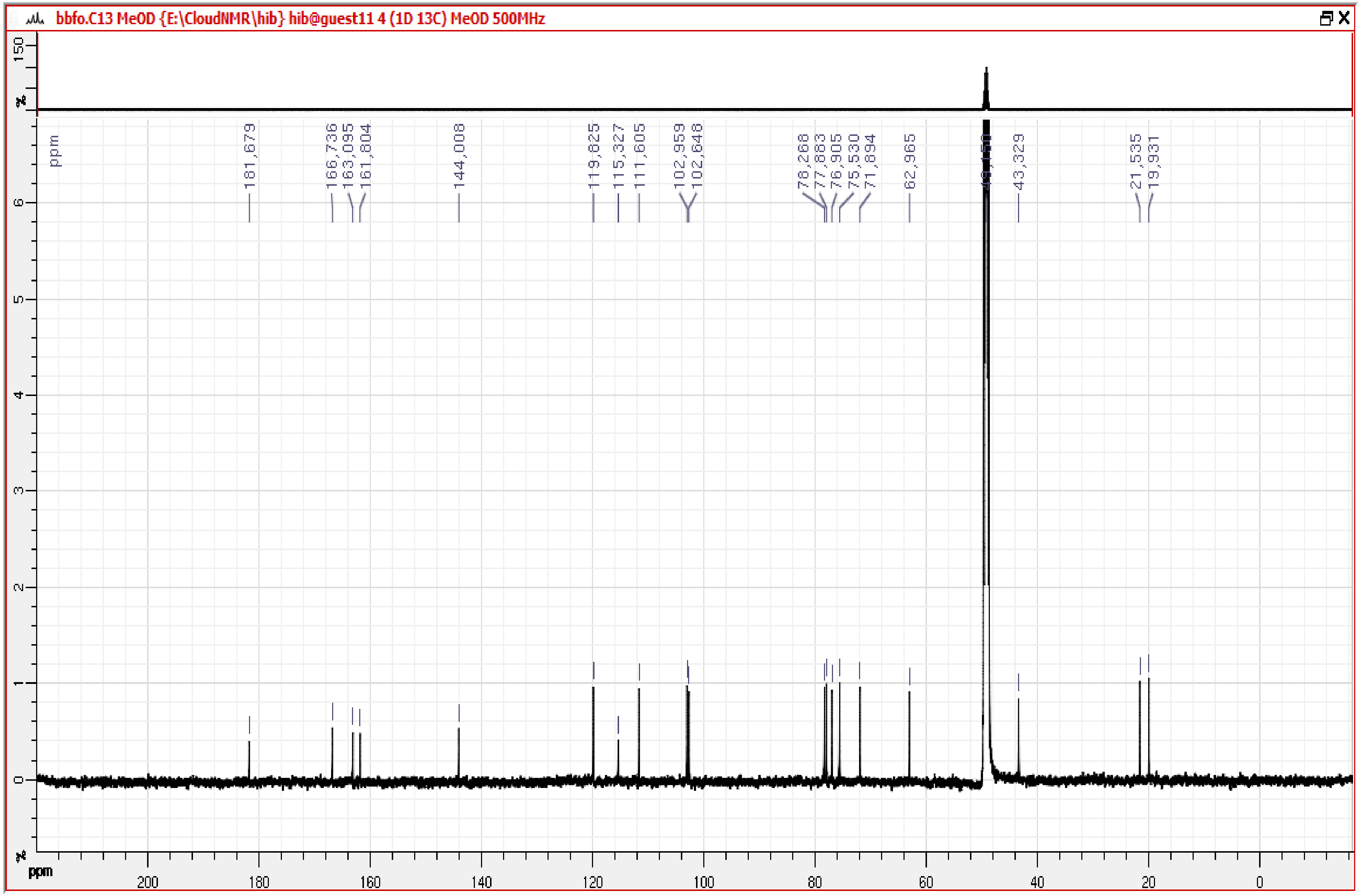

bbfo.C13 MeOD {E:\CloudNMR\hib} hib@guest11 4 (1D 13C) MeOD 500MHz
ppm
181,679
166,736
163,095
161,804
144,008
119,825
115,327
111,605
102,959
102,648
78,268
77,883
76,905
75,530
71,894
62,965
43,329
21,535
19,931
ppm
200
180
160
140
120
100
80
60
40
20
0

COSY spectral

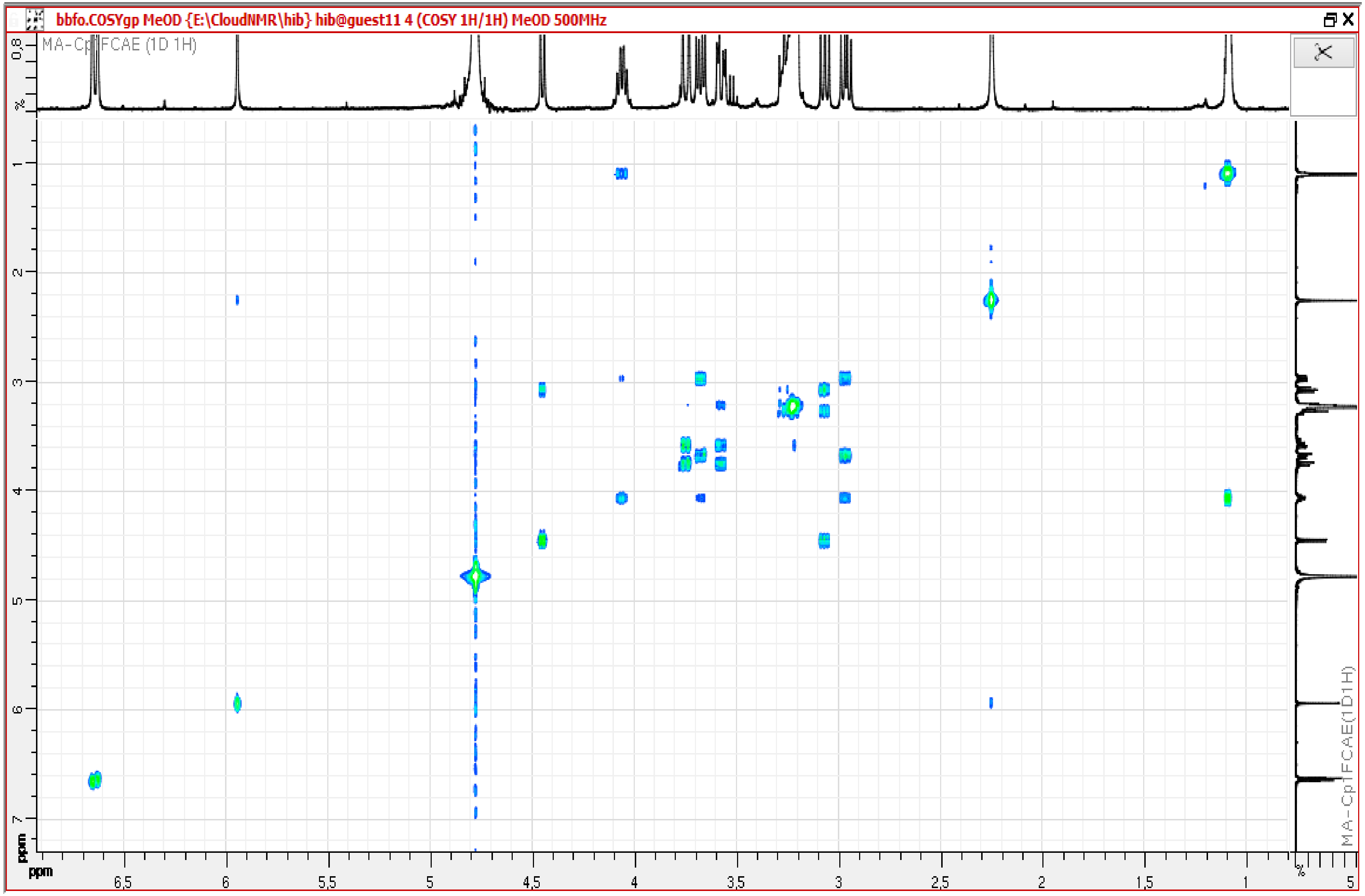

NOESY spectral

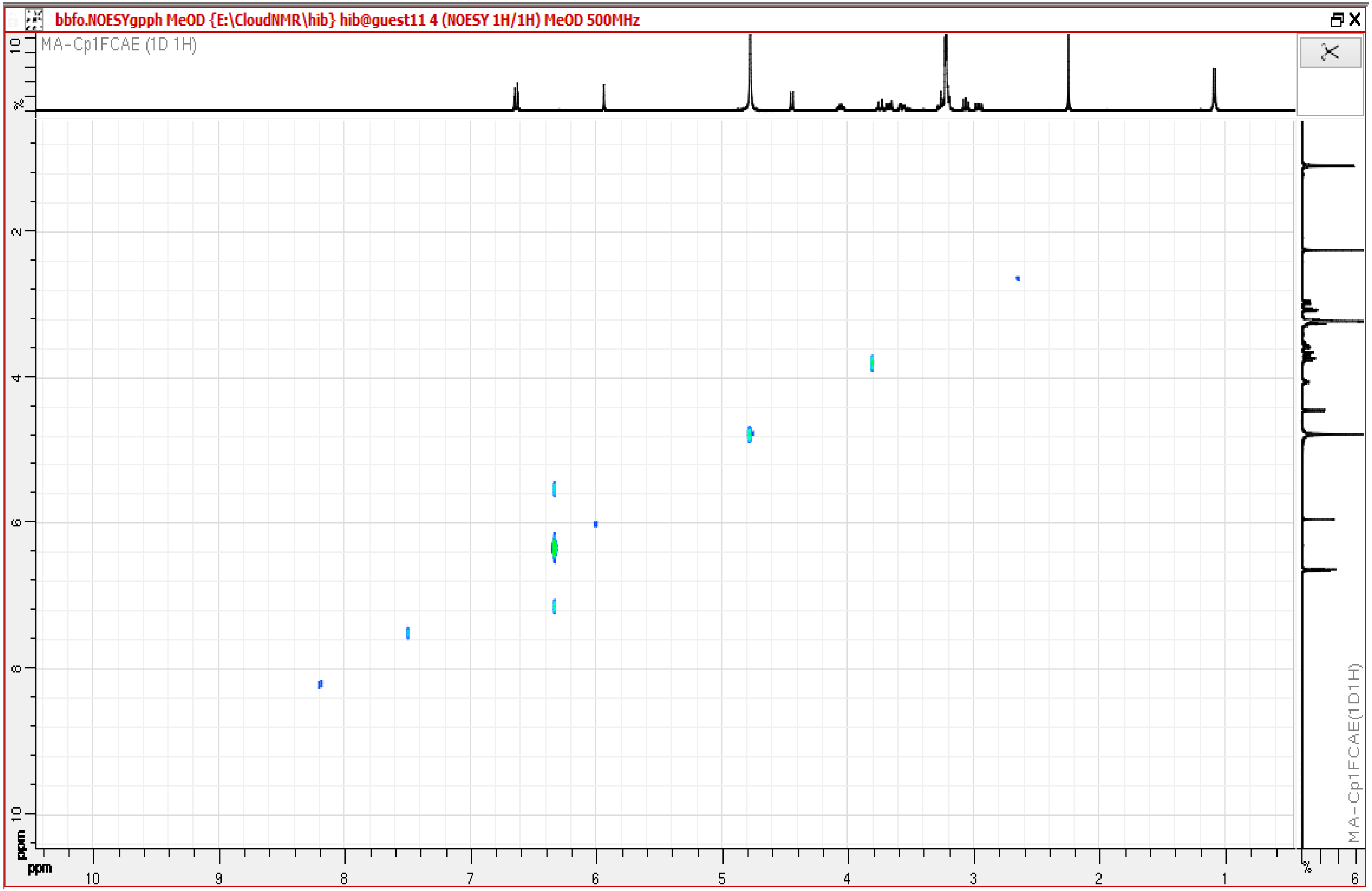

HSQC spectral

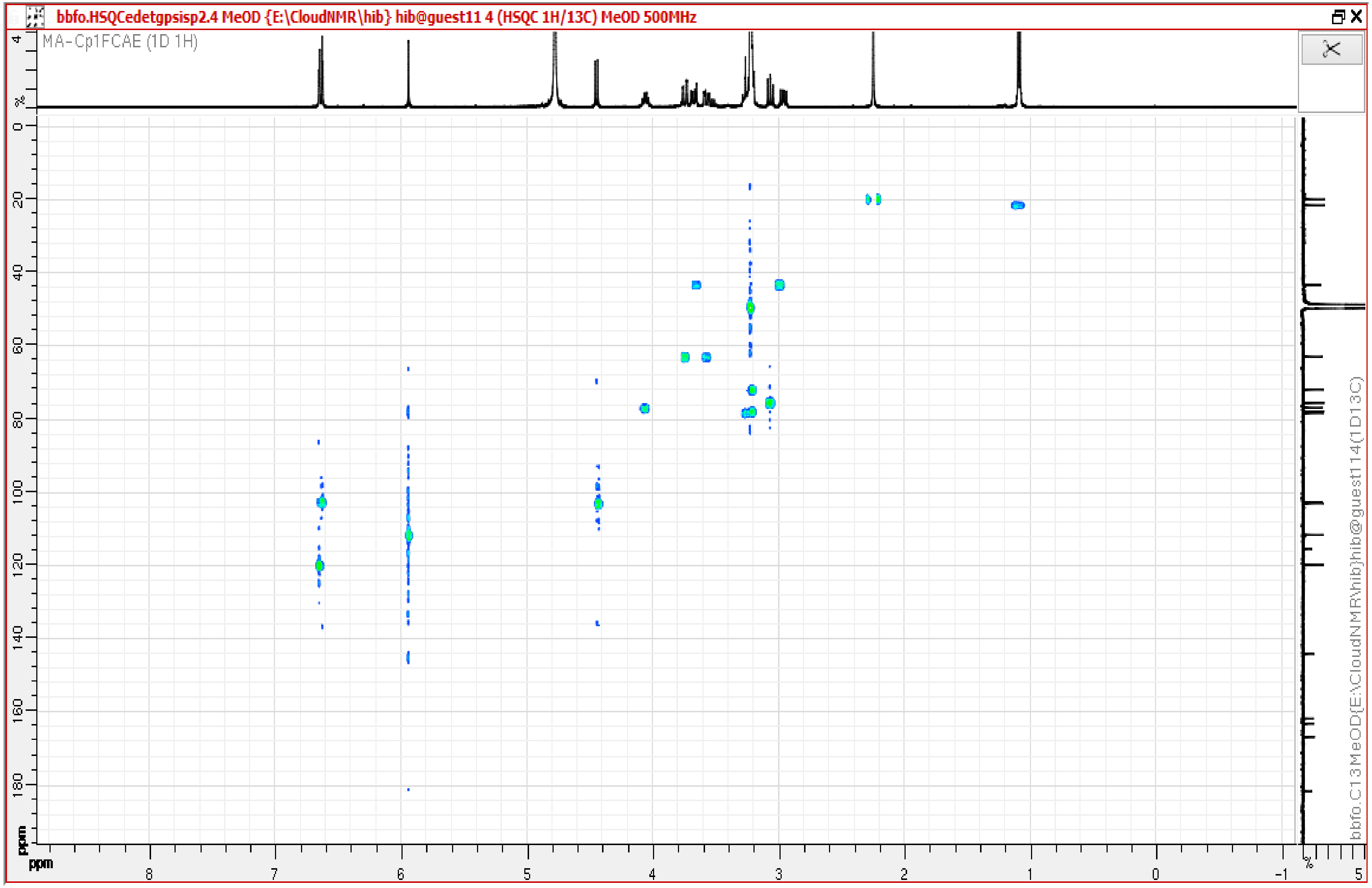

HMBC spectral

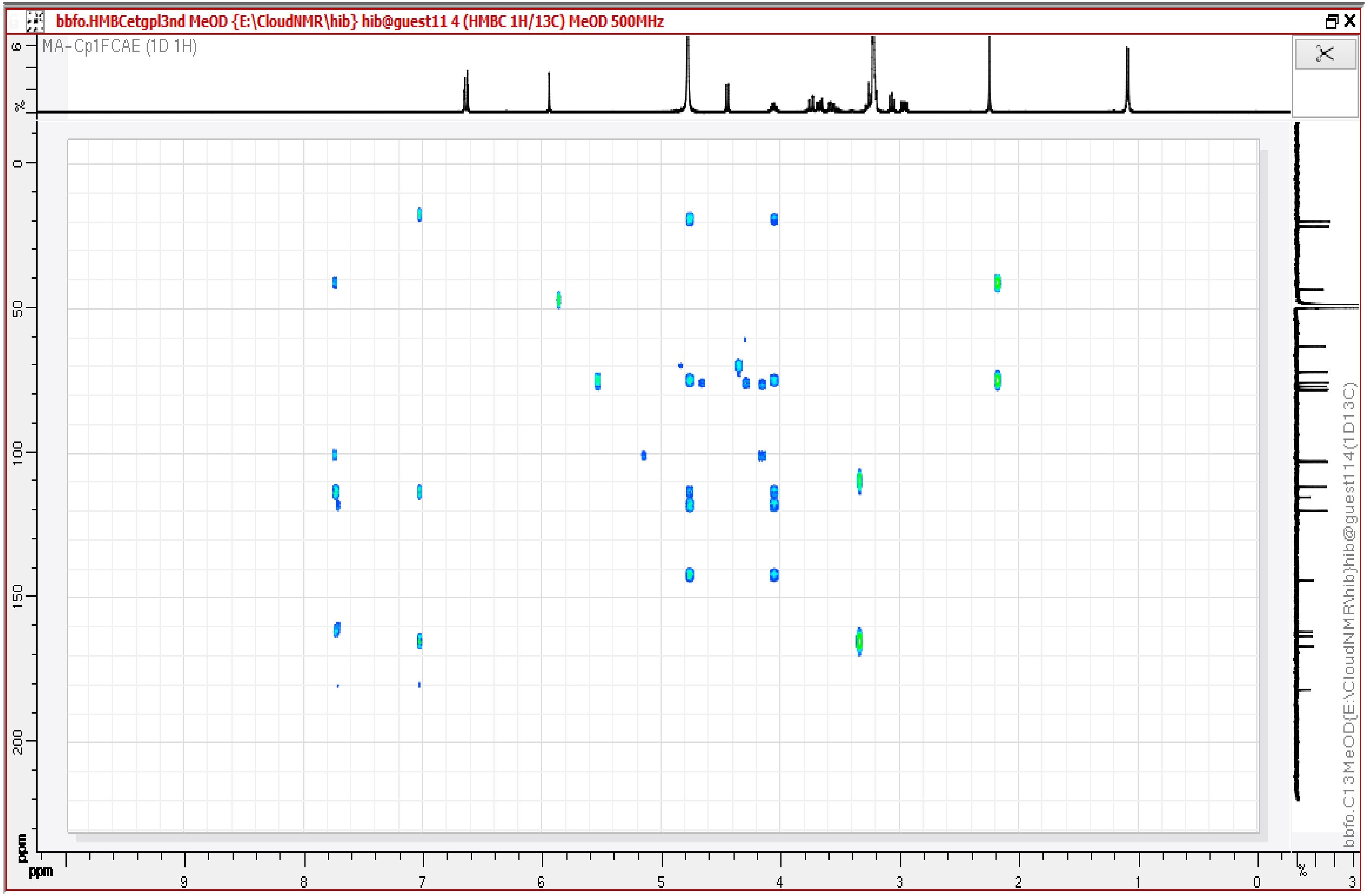

Masse spectral

Qualitative Analysis Report

**Data Filename**	QT09944.d	**Sample Name**	CP1FcAe
**Inj. Vol.**	0.1	**Position**	P1-C3
**Instrument Name**	SCA Illkirch QToF	**User Name**	PW
**Acq Method**	C18-2,1x5x1,8.m	**Acquired Time**	2/19/2015 12:09:42 PM
**IRM Calibration Status**	Success	**DA Method**	C18-2,1x5x1,8.m
**Comment**			

Sample Group **Info.**

User Chromatograms

Fragmentor Voltage 120 **Collision Energy** 0 **Ionization Mode** ESI

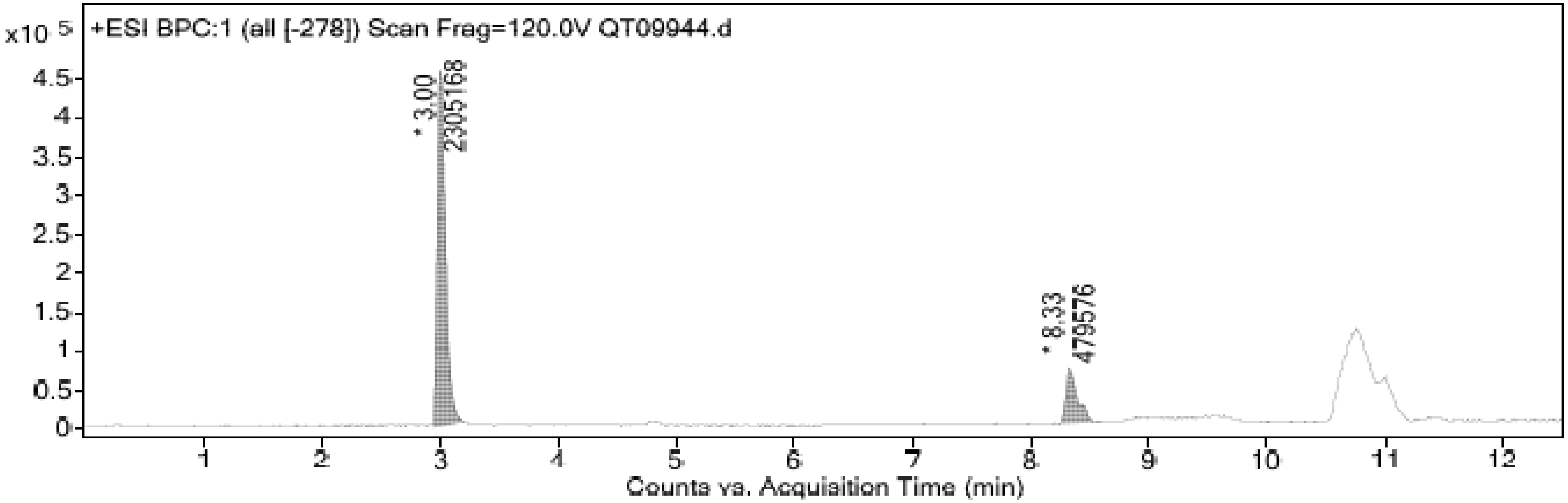

Integration Peak List

Start	RT	End	Height	Area	Area %	AreaSum%
2.94	3	3.2	460053	2305168	100	82.78
8.24	8.33	8.57	70550	479576	20.8	17.22

Qualitative Analysis Report

Spectrum Source	Fragmentor Voltage	Collision Energy	Ionization Mode
Peak (1) in "+ BPC:1 (all [-278]) Scan"	120	0	ESI

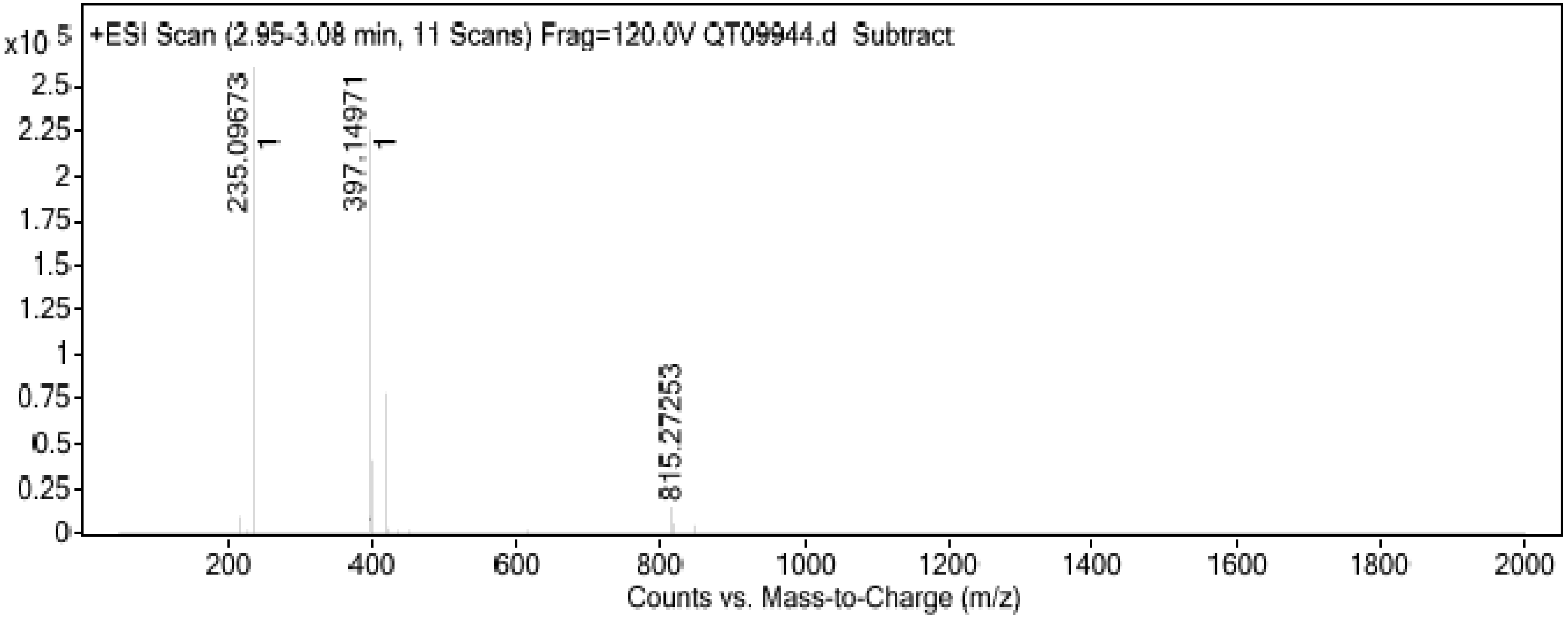

Peak List

m/z	z	Abund	Formula	Ion
235.09673	1	265350.1	C13 H15 O4	(M+H)+
236.09981	1	29850.1	C13 H15 O4	(M+H)+
397.14971	1	232350.3	C19 H25 O9	(M+H)+
398.15266	1	39968.4	C19 H25 O9	(M+H)+
419.13118		78852.9		
815.27253		13898.5		

UV spectral

Qualitative Analysis Report

Spectrum Source
Peak (1) in "DAD1 - EWC:Sig=210,8 Ref=520,8
Sub"

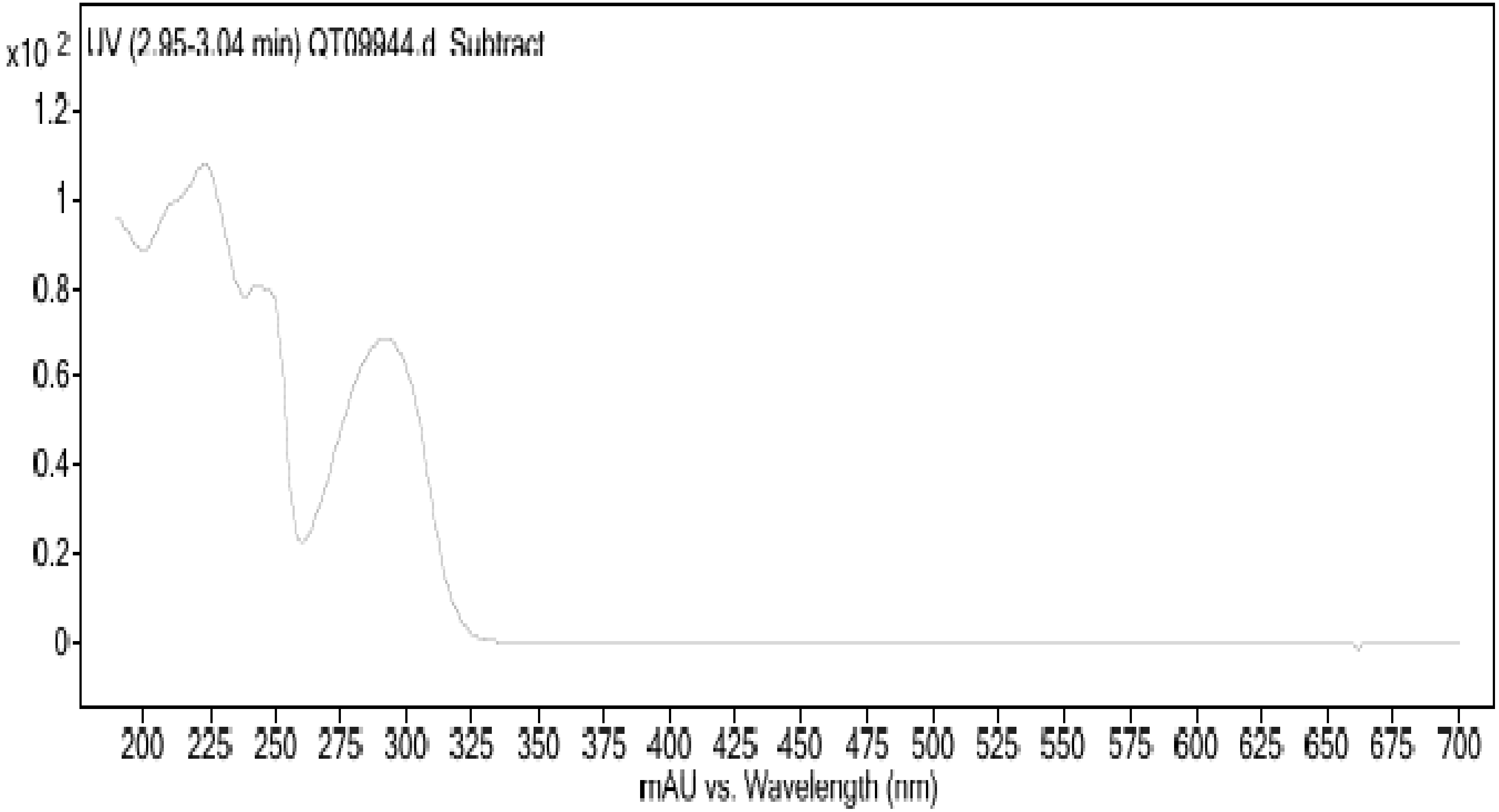

Supplement: Additional file 1: — NMR and MS spectrum of the new chromone derivative (Acthaside). (PDF 439 kb) [file 12906_2016_1489_MOESM1_ESM.pdf]
